# The transcription factor CUX1 negatively regulates invasion in castrate resistant prostate cancer

**DOI:** 10.18632/oncotarget.27494

**Published:** 2020-03-03

**Authors:** Emma R. Dorris, Amanda O’Neill, Ann Treacy, Helmut Klocker, Omri Teltsh, Elaine Kay, R. William Watson

**Affiliations:** ^1^ UCD School of Medicine, Conway Institute of Biomolecular and Biomedical Research, University College Dublin, Belfield, Dublin, Ireland; ^2^ Pathology Department, Mater Private Hospital, Dublin, Ireland; ^3^ Department of Urology, Medical University of Innsbruck, Innsbruck, Austria; ^4^ Department of Pathology, Royal College of Surgeons in Ireland, Beaumont Hospital, Dublin, Ireland

**Keywords:** castration-resistant prostatic cancer, neoplasm invasiveness, androgen-independent prostatic cancer, recurrence

## Abstract

Metastatic prostate cancer is treated with androgen ablation therapy but progress to castrate resistant prostate cancer (CRPC). This study aimed to investigate the role of CUX1 in CRPC using clinical samples and *in vitro* models. CUX1 expression was increased in androgen-independent cells compared to androgen-sensitive cells. The multi-isoform nature of CUX1 makes it difficult to assay in tissue microarrays as there is no epitope able to distinguish the many isoforms for immunohistochemistry. Using surrogate markers, we found no differential expression between castrate resistant and local hormone naïve tissue. However, differences have been demonstrated at the transcript level. In androgen-sensitive cells, migration, but not invasion, increased following CUX1 knockdown. Conversely, in androgen-independent cells, invasion was increased. This observed difference in invasion capacity is not E-cadherin mediated, as CUX1 knockdown increases the expression of E-cadherin in both cell lines with no inter-cell line difference. Cells expressed different ratios of p110/p200 isoforms depending on androgen status and cathepsin L was only detectable in androgen-sensitive cells. MMP3 is upregulated in the androgen-independent cells. Rather than a simple presence or absence of CUX1, the relative balance of CUX1 isoforms and their interplay may be a significant factor in the functional role of CUX1 in CRPC.

## INTRODUCTION

Prostate cancer is the second most common cancer in men, accounting for an estimated 14.5% of cancers diagnosed in men worldwide in 2018 [1]. Prostate cancer is the fifth leading cause of death from cancer in men; 6.6% of the total men cancer deaths worldwide [2, 3]. Localised prostate cancer is potentially curable by surgery or radiation therapy, but advanced disease represents a significant clinical challenge with no effective treatments. Proliferation of prostatic cells is dependent upon androgens and non-organ confined prostate cancer is treated with androgen ablation therapy. This can result in initial rapid responses and reduction in tumour size in men with metastatic disease. However, the disease recurs in nearly all patients within three years of treatment and the disease progresses to castrate resistant prostate cancer (CRPC), whereby the recurrent tumours no longer require androgens for growth or survival [4].

Although initially considered to be androgen-signalling independent, it is now known that CRPC can often remain hormone driven [5]. Multiple and varied mechanisms are responsible for the castrate resistant phenotype (reviewed in [6]). Prostate cancer is a heterogeneous disease and the development of CRPC is a complex process. Molecular targeting of individual genes or proteins has only had minor impact on overcoming resistance, as exemplified by clinical trials targeting MTOR in naïve CRPC [7]. Transcription factors regulate multiple signalling pathways and biological processes. As such, understanding the central transcription factors underlying the castrate resistant phenotype may provide a more appropriate therapeutic targeting approach.

Our group previously combined transcriptomic analysis with bioinformatic prediction tools to identify transcription factors associated with an experimental model of the CRPC phenotype [8]. The transcription factor cut-like homeobox 1 (CUX1) was among the predicted transcription factors associated with castrate resistance. CUX1 was chosen for further clinical and functional analysis because it is involved in cellular processes relevant to cancer including cell proliferation, cell motility and invasiveness [9–12]. There is contradictory evidence between the association of CUX1 and cancer. Most studies to date have attributed an oncogenic role for CUX1 in human cancer [11–14]. Studies in breast cancer have identified an association between elevated CUX1 expression and tumour progression and CUX1 expression was inversely correlated with relapse-free and overall survival in a small subset of breast cancer tissues [15, 16]. However, a large scale genomic analysis of 7,651 diverse human cancers identified inactivating mutations in CUX1 in 1-5% of tumours and concluded that CUX1 acts as a tumour suppressor [17]. CUX-1 has been shown to play a role in breast cancer progression and in drug resistance in gastric cancer but to date has minimal functional association with prostate cancer [11, 18, 19].

CUX1 is a multi-isoform protein (reviewed in [20]). The full length protein is approximately 200 kDa and predominantly acts as a transcriptional repressor [21]. The p200 CUX1 can be proteolytically cleaved into multiple isoforms with distinct transcriptional regulation, including the p110CUX1 isoform that acts as both a transcriptional activator and repressor [13]. CUX1 also has distinct splice variants; the p75CUX1 isoform of the transcription factor has been associated with breast cancer and myeloid leukaemia [11, 14]. In this study, we investigate the association of CUX1 in clinical samples of CRPC and investigate its role in CRPC using a preclinical model of disease.

## RESULTS

### Prostate adenocarcinoma cells express different ratios of p110/p200 isoforms depending on androgen status

**Figure 1:**
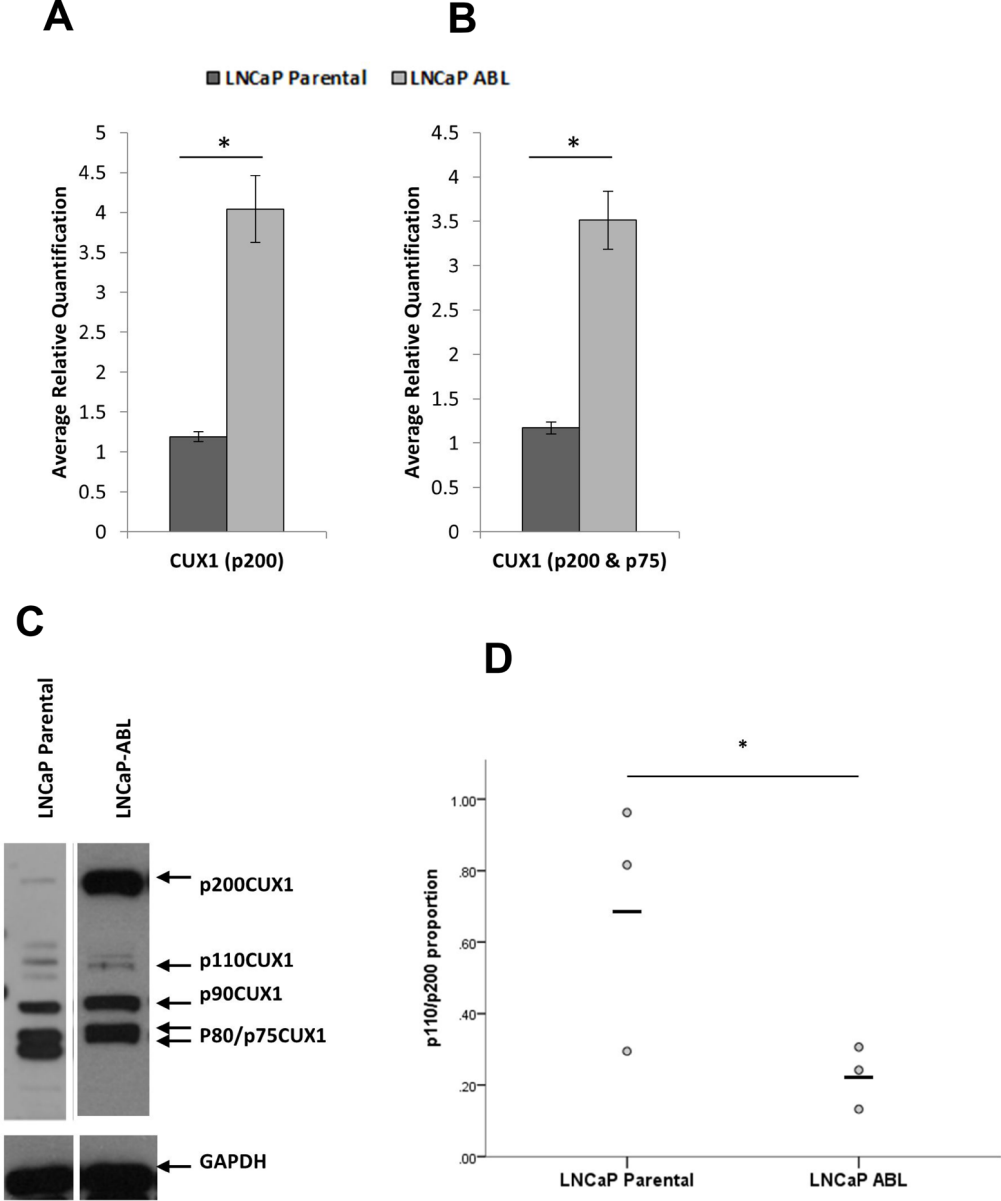
CUX1 is differentially expressed in castrate resistant prostate cancer. (**A**) Gene expression of CUX1 using probes that detect transcript variants that produce full length (p200) CUX1 only (*p* = 0.003) or (**B**) full length p200CUX1 and alternatively spliced p75CUX1 transcript (*p* = 0.002) (**C**) Protein expression of CUX1 isoforms in whole cell line lysates (**D**) Proportion of p110/p200 CUX1 isoforms (densitometry *n* = 3 independent experiments, *p* = 0.045).

The prostate adenocarcinoma cell line LNCaP is androgen sensitive whereas the derivative subline LNCaP ABL is androgen-independent [22]. CUX1 has increased gene expression in androgen-independent LNCaP-ABL compared to androgen-sensitive LNCaP (*p* = 0.003; Figure 1A). CUX1 has multiple splice variants, most notably the p75 isoform. Both p200 and p110 are processed from the same gene transcript and can be detected with a primer to exon boundary 16/17, which detects the full-length transcript only (Figure 1A). In order to determine if the p75 isoform would confound our results, we also measured CUX1 gene expression with primers that detected both the full length and alternatively spliced isoforms (primers to exon boundary 21/22). As per the full-length transcript, CUX1 was increased in the LNCaP-ABL cell line compared to LNCaP Parental (*p* = 0.002; Figure 1B). There was no significant difference in the relative gene expression levels detected by two primer sets (*p* = 0.779).

CUX1 expression is increased in LNCaP-ABL cells (Figure 1C). Densitometry of the p200 and p110 isoforms, normalised to endogenous control, demonstrates a statistically significant upregulation in the p200 (*p* = 0.028) but not the p110 (*p* = 0.362) isoform in LNCaP-ABL compared to LNCaP parental cells. The ratio of p110: p200 protein is reduced in LNCaP-ABL cells, with the androgen-sensitive LNCaP parental cells expressing a mean p110/p200 proportion of 0.69 (± 0.20) compared to 0.23 (±0.05) in the androgen-independent LNCaP-ABL (*p* = 0.04; Figure 1D). Thus, although the androgen-independent cells express more CUX1 p200, a reduced proportion of the full-length protein is being cleaved to the p110 isoform.

### Silencing of CUX1 alters cellular phenotype in prostate adenocarcinoma cells

**Figure 2:**
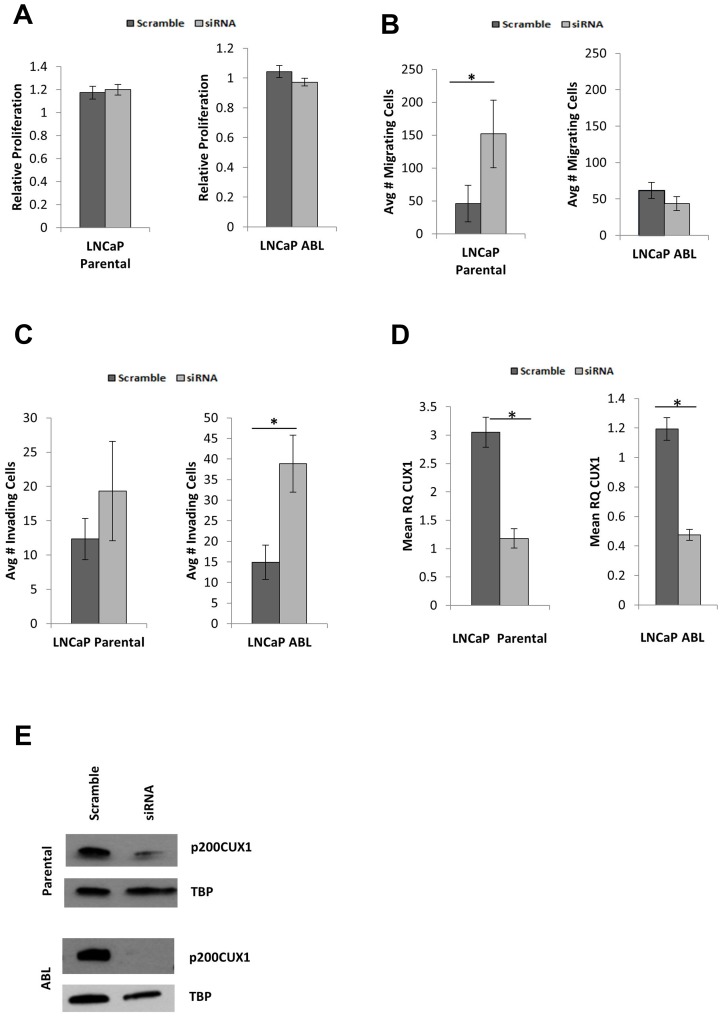
Silencing CUX1 alters cellular phenotype. (**A**) CUX1 knockdown does not affect proliferation in either cell line. (**B**) Migration is increased in response to CUX1 knockdown in LNCaP (*p* = 0.003) but not LNCaP ABL cells (*p* = 0.157) (**C**) Invasion of the castrate resistant cells LNCaP ABL is increased in response to CUX1 knockdown (*p* = 0.000) but not in the androgen sensitive parental cell line (*p* = 0.127). (**D**) Representative knockdown of CUX1 at gene and (**E**) protein levels. Graphs are mean of *n* = 3 independent experiments ± SEM.

Knockdown of CUX1 does not alter cell proliferation in LNCaP cells. CUX1 was knocked down with a non-targeting siRNA used as control. No difference was observed in the proliferative capacity of LNCaP parental cells following knockdown of CUX1 (*p* = 0.687; Figure 2A). Similarly, no difference was identified in the proliferation of LNCaP-ABL cells following CUX1 knockdown (*p* = 0.829; Figure 2A). For all knockdown experiments, CUX1 knockdown was confirmed by gene expression analysis (Figure 2D), with CUX1 significantly downregulated in siRNA-treated cells compared to scramble control (*P* < 0.05) for all assays. Knockdown was also confirmed by Western blot analysis of p200CUX1 (Figure 2E).

### CUX1 knockdown modifies migration and invasion capacity of cells

Prior to transfection, the migration rate between LNCaP parental and LNCaP-ABL cells were measured. No significant difference was observed between LNCaP parental (mean 46.33 ± 27 cells per field) and LNCaP-ABL cells (mean 61.78 ± 11.15 cells per field), *p* = 0.516. When CUX1 was knocked down, there was a statistically significant increase in the number of cells migrating through the transwell inserts in LNCaP parental (*p* = 0.003) but not LNCaP-ABL (*p* = 0.126) cells (Figure 2B).

Prior to transfection, the invasion rate between LNCaP parental and LNCaP-ABL cells was measured. No significant difference was observed between LNCaP parental (mean 12.33 ± 3.01 cells per field) and LNCaP-ABL cells (mean 14.89 ± 4.20 cells per field), *p* = 0.728. In response to CUX1 knockdown, no significant difference was observed in the androgen sensitive LNCaP cells (*p* = 0.157). However, in the androgen-independent LNCaP-ABL cells the average number of invading cells was greater than 2.5-fold increased following knockdown of CUX1 (Figure 2D; *p* < 0.001). Relating the invasion rates to the migratory rates of the cells, in LNCaP and LNCaP-ABL the non-targeting (scramble) transfection control had 26.6% and 24.1% invading cells compared to migrating cells respectively. Upon CUX1 knockdown, LNCaP parental had invasion rate of 12.72% (0.48 compared to scramble control); whereas LNCaP-ABL had an invasion rate of 88.83% (3.69-fold greater than scramble control).

### Surrogate markers of CUX1 are not differentially expressed in clinical prostate cancer tumour tissue samples

CUX1 has multiple isoforms and, as such, detection of specific isoforms presents significant challenges. The sequence of the CUX1 isoforms makes it extremely difficult to detect the specific isoform of interest in IHC analysis. The p200CUX1 protein shares the N-terminus with the dominant negative, non-DNA binding p150CUX1 cleavage product and the (non-transcription factor) CASP isoform. Therefore, N-terminal epitopes are not p200CUX1 specific. At least one cleavage product isoform, which can have distinct transcriptional regulation, will overlap with p200CUX1 [20, 23] and there are no p200CUX1-specific epitopes. It has previously been reported that IHC staining with CUX1 antibodies was not sensitive enough to detect expression of the endogenous CUX1 proteins in mice [11]. During our optimization of CUX1 antibodies for IHC, cytoplasmic but not nuclear staining was detectable (data not shown). As the p200CUX1 and p110CUX1 isoforms were readily detectable by Western blot in nuclear fractions at high levels, it could not be concluded with confidence that the observed staining was representative of p200CUX1. Therefore, surrogate markers were employed for IHC analysis of clinical samples.

### FTO and MARCKS as surrogate markers for CUX1

**Figure 3:**
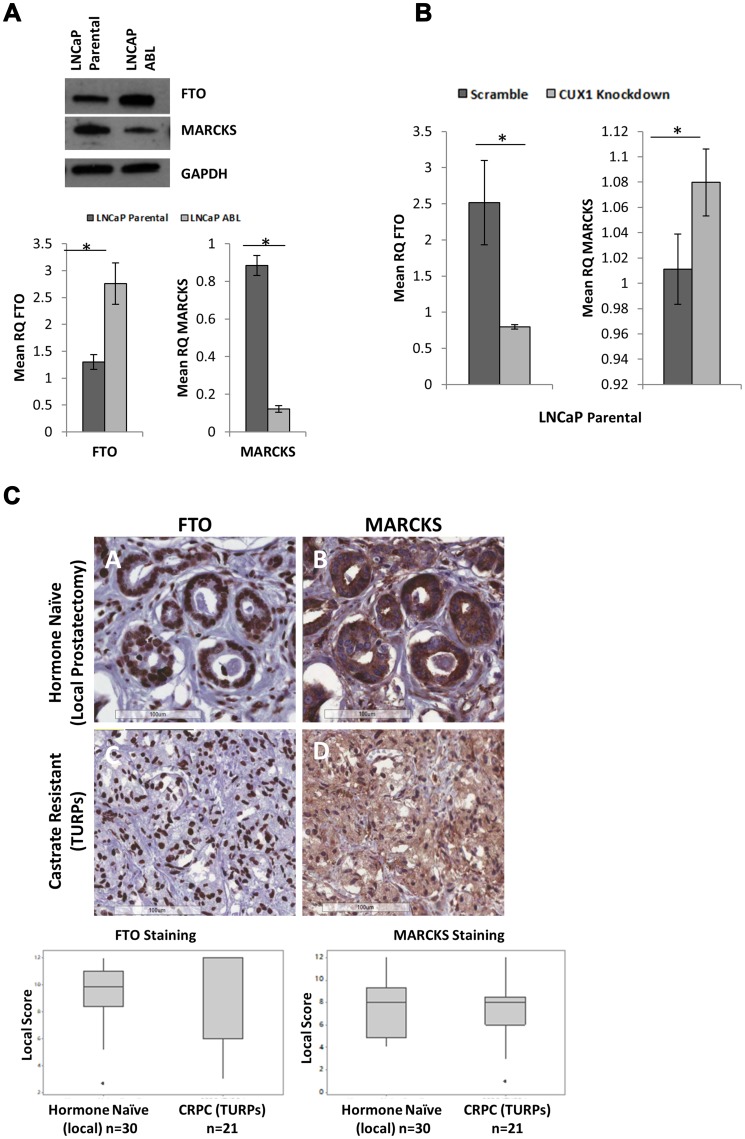
Surrogate Markers of CUX1 are not differentially expressed in hormone naïve and castrate resistant prostate cancer. (**A**) FTO and MARCKS are differentially expressed in LNCaP parental and ABL cells at both the protein and messenger level. (**B**) Knockdown of CUX1 in LNCaP parental cells decreases FTO gene expression and increases MARCKS gene expression (*p* < 0.05). (**C**) Representative staining of FTO (nuclear) and MARCKS (cytoplasmic). Magnification 200 ×. Boxplots based on Loda scoring of FTO and MARCKS staining between castrate resistant TURPs TMA and hormone naïve (local prostatectomy) TMA. No significant difference was found (One-Way ANOVA α = 0.05).

**Figure 4:**
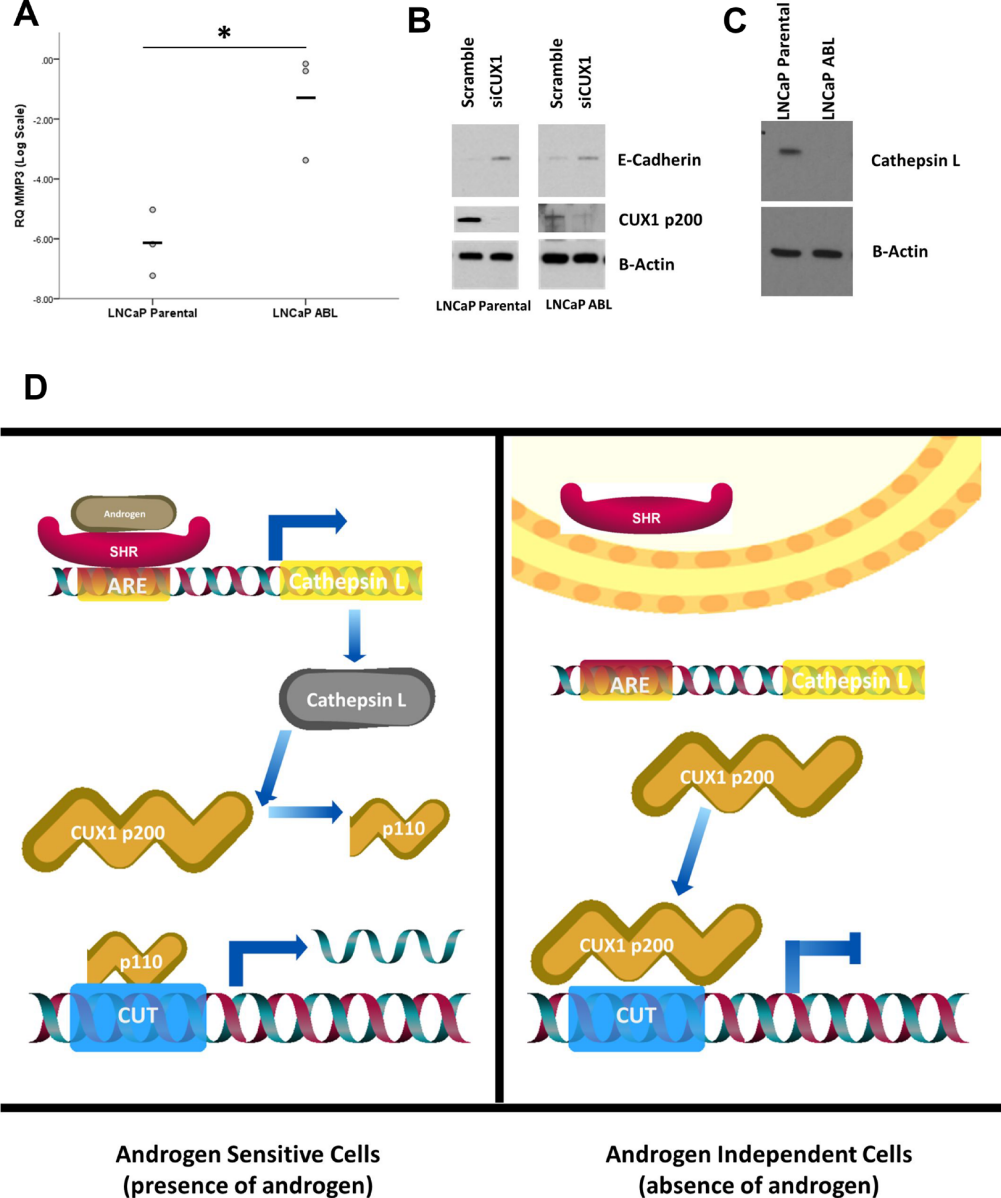
Phenotypic differences in response to CUX1 silencing are driven by androgen status. (**A**) The androgen-independent cell line LNCaP ABL has a higher expression of MMPs compared to androgen-dependent LNCaP parental (*p* = 0.046). (**B**) CUX1 knockdown induces expression of E-Cadherin in both cell lines (**C**) Cathepsin L, the enzyme response for cleavage of p200 to p110 is expressed in androgen-dependent LNCaP only. (**D**) Schematic showing androgen-dependent regulation of cathepsin L. Cathepsin L contains an androgen response element (ARE) and is regulated by the androgen receptor. In the presence of androgen cathepsin L is expressed and cleaves p200 to p110, increasing the ratio of p110: p200. This does not occur in the absence of androgen, skewing the balance of CUX1 to p200 dominant.

Our laboratory has previously published a gene chip experiment between LNCaP parental and LNCaP-ABL cell lines [8]. Differentially regulated genes between cell lines that were bioinformatically predicted to be regulated by CUX1 were cross referenced with the literature on validated CUX1 interactants. This identified fat mass and obesity associated gene (FTO) [24], which was upregulated, and myristoylated alanine-rich protein kinase C substrate (MARCKS) [25], which was downregulated in our previous gene chip analysis in LNCaP-ABL compared to LNCaP parental cells. This pattern of gene expression was confirmed for both FTO (*p* = 0.002) and MARCKS (*P* < 0.001; Figure 3A). Furthermore, the pattern of expression translated to the protein level, with FTO increased and MARCKS decreased in LNCaP-ABL compared to LNCaP parental cells (Figure 3A). The relative expression of these genes was assessed in LNCaP parental cells following CUX1 knockdown (Figure 3B). Knockdown of CUX1 was associated with a significant decrease in FTO expression and a small but statistically significant increase in MARCKS expression (Figure 4D). These results suggest that, as expected, CUX1 predominately acts as a negative regulator of MARCKS and a positive regulator of FTO transcription.

### Surrogate markers of CUX1 are not differentially expressed in clinical prostate cancer tissue microarrays

Tissue microarrays containing 21 trans urethral resection of the prostate (TURPs) castrate resistant (Austrian) patient samples and 30 local prostatectomy hormone naïve (Irish) patients were assessed for the surrogate markers FTO and MARCKS. If CUX1 was overexpressed in CRPC, as found in the cell line model, FTO should be upregulated and MARCKS should be downregulated in the CRPC (TURPs) TMA compared to the hormone naïve (local) TMA. FTO had nuclear staining and MARCKS had cytoplasmic staining, as expected (Figure 3C). Immunohistochemical staining was scored by an independent pathologist and analysed using both the Loda and Allred scoring systems [26, 27]. No significant difference was observed between TMAs for either FTO or MARCKS (Figure 3C), irrespective of scoring system used.

### Phenotypic differences in response to CUX1 silencing are driven by androgen status

#### MMP3 is increased in androgen-independent cells

The extracellular matrix digesting matrix metalloprotease (MMP) family of proteins have previously been shown to be regulated by the androgen receptor [28]. Therefore, we assayed the expression of MMP3 in LNCaP parental and LNCaP-ABL cells. MMP3 gene expression was higher in the androgen-independent LNCaP-ABL cells compared to androgen-sensitive LNCaP Parental cells (*p* = 0.016, Figure 4A). This increase in MMP3 likely accounts, at least in part, for the increased invasive rate of LNCaP-ABL compared to LNCaP parental cells observed in Figure 2C.

### CUX1 knockdown increases E-cadherin

E-cadherin is an adhesion protein of the classic cadherin superfamily. E-cadherin has an established regulatory relationship with both the p110 CUX1 isoform and androgen signalling [25, 29]. We measured the protein expression of E-cadherin in response to CUX1 knockdown. Pre-transfection, there was no significant difference in baseline density measurements of E-cadherin between LNCaP parental and LNCaP-ABL cells (*p* = 0.148). CUX1 knockdown increased the expression of CUX1 in both cell lines (Figure 4B), with no significance inter-cell line difference observed in the density of expression between LNCaP and LNCaP-ABL cells (*p* = 0.403). Thus, although CUX1 regulates E-cadherin, our data indicates that the difference observed in invasion capacity between the two cell lines is not mediated by E-cadherin.

### Cathepsin L expression is reduced in androgen-independent cells

Cathepsin L is the proteinase responsible for the processing of CUX1 p200 into the p110 isoform [24]. As the androgen-sensitive LNCaP parental cell line has a higher p110/p200 ratio compared to androgen-independent LNCaP-ABL (Figure 1D), we assayed the expression of cathepsin L in both cell lines. Cathepsin L is readily detectable in LNCaP parental cells but not in the LNCaP-ABL subline (Figure 4C). Analysis of cathepsin L identified multiple androgen response elements (AREs) in cathepsin L (*CTSL*) including both full and half-site AREs. Cathepsin L is positively regulated by both full and half site AREs [30]. As such, we hypothesize that the observed differences observed between androgen-sensitive and androgen-independent cells in response to CUX1 knockdown is mediated by cathepsin L and the relative levels of p200/p110 CUX1 (Figure 4C). In androgen sensitive LNCaP cells, cathepsin L cleaves p200 to the p110 isoform at a high rate (see Figure 1D). As CUX1 p200 and p110 can have distinct transcriptional properties and DNA binding affinities [21], the androgen-independent cell line LNCaP-ABL has less CUX p110-mediated response.

## DISCUSSION

Systemic androgen-deprivation therapy (ADT) is the therapeutic mainstay to treat men with metastatic prostate cancer. ADT is based on the dependency of prostate cells for androgens to grow and survive. The inability of ADT to effectively eliminate all metastatic prostate cancer cell populations is manifested by inevitable relapse (CRPC). CRPC is an extremely heterogeneous disease that affects patients with varying metastatic burden and symptoms. This heterogeneity results in variable survival estimates ranging from months to several years [31]. Understanding the biology of castrate resistance in prostate cancer is crucial for improved treatment and disease outcomes. In this study we have demonstrated that androgen-sensitive cells express cathepsin L and higher relative levels of the p110 isoform of CUX1, whereas androgen-independent cells have lost cathepsin L expression and express higher relative levels of uncleaved p200 CUX1. The p200CUX1 isoform acts as a repressor of transcription whereas the amino-terminally processed p110CUX1 isoform has a dual role as a transcriptional activator and repressor [32]. Thus, rather than a simple presence or absence of CUX1, the relative balance of CUX1 isoforms and their interplay is a significant factor in the functional role of CUX1 in castrate resistant prostate cancer.

The ability to migrate and invade into surrounding tissues is a prerequisite for local tumour progression and disease advancement. Several previous studies in other cancer types have identified a role for CUX1 in mediating tumour progression via its role in cell migration and invasiveness [15, 19, 25, 33]. Most of these studies focused on the p110 isoform and found an E-cadherin-mediated mechanism. Our work has demonstrated that CUX1 knockdown increases migration in androgen-sensitive cells and invasion in androgen-independent cells. E-cadherin expression was increased in both cell lines in response to CUX1 knockdown, confirming the previously identified feedback loop between CUX1 and E-cadherin; but this does not explain the differences observed in response to CUX1 knockdown between androgen-sensitive and androgen-independent cells.

The phenotypic difference observed in response to CUX1 knockdown between the two cell lines may be due, at least in part, to the androgen status of the cell lines. The invasion associated matrix metalloprotease MMP3 contains a full ARE and has previously been shown to be downregulated via the androgen receptor [28, 30]. Here, we demonstrate that MMP3 is upregulated in the androgen-independent LNCaP-ABL cell line compared to LNCaP parental cells. Thus, the mechanism underlying the increase in invasion observed in response to CUX1 knockdown in the androgen-independent LNCaP-ABL cell is in part mediated by increased MMP3 in androgen-independent cells.

CUX1 has been described as an important mediator of a transcriptional regulatory cascade involved in cell migration and epithelial-to-mesenchymal transition (EMT) [19]. P110 CUX1 functions to activate the expression of SNAIL, which in turn co-operates with CUX1 to regulate downstream targets involved in migration [25]. This function of CUX1 is specific to the p110 isoform. Burton *et al.* recently demonstrated in mesenchymal prostate cancer cells that this effect could be abrogated via inhibition of cathepsin L. Cathepsin L contains both full and half-site AREs, both intronic and upstream of its transcriptional start site and its expression could not be detected in the androgen-independent cell line. This lack of cathepsin L is reflected by the reduced proportion of p110 relative to p200 isoform observed in the androgen-independent cell line.

As outlined earlier, there is no epitope unique to, and thus no antibody specific to, the p200CUX1 isoform. p200CUX1 was abundantly detectable in nuclear extracts by western blotting. However, during the optimization of IHC to detect CUX1, cytoplasmic staining was observed with little nuclear staining. Western blotting has the benefit of size to identify the isoform of interest; however, this is not possible in immunohistochemical analysis. To circumvent these issues in detecting p200CUX1, IHC analysis of surrogate markers was employed. By using markers differentially regulated by p200CUX1 (FTO positively regulated and MARCKS negatively regulated), we hypothesized that an observed increase of FTO and corresponding decrease of MARCKS would be indicative of an up regulation of CUX1. We confirmed that expression of these markers is altered following CUX1 knockdown in our cell line models. The limitation of this method to only provide indirect evidence of p200CUX1 status is acknowledged. That no significant difference was observed in the surrogate markers does not preclude an alteration of CUX1 in clinical CRPC. This demonstrates that the indirect immunohistological marker approach we used did not correlate in our clinical samples. As protein is more stable than RNA, they are preferred biomarkers. Here we highlight that CUX1 may be unsuitable as a protein biomarker. Our findings are further supported by data from the pathology atlas of the human cancer transcriptome [34] in combination with the Human Protein Atlas [35] that reports CUX1 protein expression is mainly not consistent with CUX1 RNA expression data. Thus, CUX1 RNA rather than protein may have biomarker potential.

Recently, Sharma *et al.* 2018 studied the association of transcription factors with Androgen Deprivation Therapy (ADT) response and metastatic progression in prostate cancer by preforming whole transcriptome analysis of 20 patient-matched Pre-ADT biopsies and 20 Post-ADT prostatectomy specimens [36]. Hierarchical clustering and principal component analysis were used to classify the samples in to two subgroups of patients (high impact and low impact groups) that exhibited distinct transcriptional changes in response to ADT. Further computational analyses identified transcription factor coordinated groups (TFCGs) enriched in the high impact group regulatory network. These TFCGs demonstrated association with pronounced initial transcriptional response to ADT, aggressive signatures, and metastasis. CUX1 has emerged as one of the key transcription factors in the high impact group network that make up these TFCGs.

We have demonstrated that CUX1 is differentially expressed in androgen-sensitive and androgen-independent prostate cancer cells. Silencing CUX1 in these cell lines have different phenotypic effects. The observed differences arise from the interplay between androgen responsive genes (MMP3 and Cathepsin L) and differential expression of the CUX1 isoforms. Thus, rather than a simple presence or absence of CUX1, the relative balance of CUX1 isoforms and their interplay may be a significant factor in the functional role of CUX1 in castrate resistant prostate cancer and may be an avenue for future work.

## MATERIALS AND METHODS

### Cell culture

The LNCaP parental prostate cancer cell line was originally obtained from the American Type Culture Collection (ATCC) and authenticated via short tandem repeat (STR) DNA profiling and comparison to the ATCC STR profile database. LNCaP parental cell lines were maintained in advanced RPMI-1640 medium supplemented with 10% FBS, 100 μl/ml streptomycin, 100 U/ml penicillin and 1% HEPES. The LNCaP-ABL androgen-independent subline was generated as described previously [22] and maintained in Advanced RPMI-1640 medium supplemented with 10% charcoal-stripped FBS, 100 μl/ml streptomycin, 100 U/ml penicillin and 1% HEPES. Cells were maintained in a humidified incubator at 37° C with 5% CO_2_. LNCaP ABL lines were authenticated via STR profiling and comparison to ATCC STR database (88% match to LNCaP ATCC number CRL-1740).

### Real-time PCR

RNA was extracted from cell line pellets using the Nucleospin miRNA extraction kit (Macherey Nagel, Germany) as per manufacturer’s instruction. RNA was quantified using the Nanodrop 2000 (Thermo-Fisher, DE, USA) and reverse transcribed to cDNA as previously described [37]. Relative quantification gene expression analysis was performed using TaqMan gene expression assays (Applied Biosystems, CA, USA, FTO probe Hs1057145_m1, MARCKS probe Hs00158993_m1, MMP3 probe Hs00968305_m1 & CUX-1 probe Hs1064021_m1) on the AB 7900HT Sequence Detection Systems (Applied Biosystems, CA, USA). Human GAPDH or eukaryotic 18s rRNA endogenous controls (Applied Biosystems, CA, USA) were employed. All samples were run in technical triplicate for *n* = 3 independent experiments.

### Western blot analysis

Whole cell protein lysates were extracted as previously described [38]. Nuclear and cytosolic fractions were extracted from fresh cell pellets washed in PBS, lysed in ice-cold cytosolic buffer (10 mM HEPES pH8.0, 1.5 mM MgCl_2_, 10 mM KCL, 200 mM sucrose, 0.25%NP40, 5 mM DTT, 1× protease inhibitor cocktail (Sigma-Aldrich, MO, USA), 1× phosphatase inhibitor cocktail 1 (Sigma-Aldrich, MO, USA)), harvested to tubes and incubated at 4° C with shaking for 30 minutes followed by centrifugation at 14,000 rpm × 10 mins at 4° C. Supernatant containing cytosolic protein fraction were kept on ice and pellets washed with ice-cold PBS. Ice-cold nuclear lysis buffer (10 mM HEPES pH8.0, 420 mM NaCl, 400 nM EDTA, 1.5 mM MgCl2, 25% glycerol, 5 mM DTT, 1× protease inhibitor cocktail (Sigma-Aldrich, MO, USA), 1× phosphatase inhibitor cocktail 1 (Sigma-Aldrich, MO, USA)) was added to pellets, incubated on ice for 30 minutes followed by centrifugation at 14,000 rpm × 30 mins at 4° C. The supernatant containing the nuclear fraction was kept on ice. Protein concentrations were determined using Bradford assay (Bio-Rad, CA, USA). Western blots were carried out as previously described [37]. The following primary antibodies were used at 1:1000 dilution unless otherwise stated: anti-CUX1 (Santa-Cruz sc-13024), anti-FTO (Santa-Cruz sc-271713), anti-MARCKS (AbCam ab52616), anti-TATA binding protein (TBP) (AbCam ab52616; 1:10000), anti-cathepsin L (Santa-Cruz sc-32320), anti-E-Cadherin (BD Biosciences 610181), anti-B-Actin (Sigma A5316, 1/5000) and anti-GAPDH (Cell Signaling Technologies 2118s). Densitometry was assed using ImageJ.

### Small-interfering RNA transfection

Cells were seeded into poly-D-lysine coated 6-well plates at 2.5 × 10^5^ cells per well and incubated for 48 hours. siRNA transfection was performed as previously described [37] using 10 nM siGENOME SMART pool targeting CUX1 or Non targeting siRNA (Dharmacon, CO, USA) as a transfection control. Effective protein knockdown was observed at 48 hours.

### Cell proliferation assay

Cells were seeded into 96-well plates at 7.5 × 10^3^ cells per well and incubated for 48 hours. Cells were transfected with siRNA and incubated for 48 hours. The Cell Proliferation Kit I (MTT) (Roche, Switzerland) was employed to measure the growth rates of proliferation. A control with no treatment added was included on all plates and used to normalize results as previously described [39].

### Migration and invasion transwell assay

Cells were seeded into poly-D-lysine coated 6-well plates at 2.5 × 10^5^ cells per well and incubated for 48 hours. Cells were transfected with siRNA and incubated for 24 hours. Cells were trypsinised and each well resuspended in 1ml of serum-free media. 300 μl cells were added to the chambers of uncoated (migration) or Matrigel-coated (invasion) transwell chambers sitting in 24-well plates as previously described [37]. 500 μl of full media was added per lower well to create a serum gradient and incubated for 48 hours. Cells were removed from the upper chamber using cotton swabs dampened in PBS and chambers were stained in 0.25% crystal violet [40]. Cells were counted from five fields at 10× magnification: one random field from each quarter and a central field. Cells from each field were added together to calculate total number of invading cells.

### Immunohistochemistry and TMA

Immunohistochemical (IHC) staining and scoring was performed as previously described [8]. Primary antibodies used were anti-FTO (Santa-Cruz sc-271713, 1:150 dilution) and anti-MARCKS (AbCam ab52616, 1:300 dilution). Two TMAs were scored. A TMA containing tissue samples of CRPC tumors obtained from 21 patients undergoing palliative transurethral resection of the prostate, as previously described [8]; and a TMA containing tumour cores from 30 patients undergoing radical prostatectomy [41]. Investigation has been conducted in accordance with the ethical standards and according to the Declaration of Helsinki and to national and international guidelines. The study was approved by the authors’ institutional review board. Immunoexpression was determined by an independent pathologist and immunoscore calculated via the Loda method [26].

### Data analysis

Data was analyzed using Minitab statistical software version 17. All data was tested for parametric assumptions. Parametric data was analyzed using 2-tailed Students’ *T*-Tests (type 1 for paired analysis, type 2 for group analyses) or One-Way ANOVA with Tukey’s post-hoc test for honest significant difference as appropriate. Nonparametric data was analyzed using a Kruskal–Wallis *H* test. An alpha level of 0.05 was used for all analysis. *P* values are shown to 3 decimal places. Assays were performed using technical triplicate for *n* = 3 independent assays. Data is presented as mean ± standard error of the mean (SEM).

## Abbreviations

ADT: androgen-deprivation therapy
ANOVA: analysis of variance
CASP: CUT alternatively spliced protein
CRPC: castrate resistant prostate cancer
CUX1: cut-like homeobox 1
EMT: epithelial-to-mesenchymal transition
FTO: fat mass and obesity associated gene
IHC: immunohistochemistry
MARCKS: myristoylated alanine-rich protein kinase C substrate
SNP: single nucleotide polymorphism
TBP: TATA binding protein
TURP: transurethral resection of the prostate
